# Early and severe cognitive impairment in multiple sclerosis

**DOI:** 10.1590/S1980-57642012DN06010008

**Published:** 2012

**Authors:** Maria Fernanda Mendes, Alessandro Finkelsztejn, Sidney Gomes, Yára Dadalti Fragoso

**Affiliations:** 1 MD, MSc, PhD, Department of Neurology, Medical School of Santa Casa São Paulo, São Paulo SP, Brazil.; 2 MD, MSc, Department of Neurology, Hospital de Clínicas de Porto Alegre, Porto Alegre RS, Brazil.; 3 MD, PhD, Department of Neurology Beneficencia Portuguesa Hospital and Paulistano Hospital, São Paulo SP, Brazil.; 4 MD, MSc, PhD, Head of the Department of Neurology, Medical School Universidade Metropolitana de Santos, Santos SP, Brazil.

**Keywords:** multiple sclerosis, cognition, dementia, basal ganglia, brain atrophy

## Abstract

**Objectives:**

To report on four new cases of severe cognitive impairment in the early stages of multiple sclerosis (MS) and to review data on the subject since few cases have been reported worldwide.

**Methods:**

Retrospective evaluation of medical records of patients with severe cognitive impairment within the first five years of MS diagnosis. Results on neuropsychological tests and magnetic resonance imaging (MRI) were disclosed.

**Results:**

Four patients from different Brazilian neurological departments in Brazil were evaluated, all presenting with severe cognitive dysfunction classified as rapidly developing dementia. MRI images showed severe brain atrophy and basal ganglia lesions in all patients.

**Conclusions:**

Although rare, severe cognitive impairment in MS represents an important disability and may ultimately constitute another form of the disease.

## Abstract (pt)

**Objetivos:**

Relatar quatro novos casos de grave comprometimento cognitivo nas fases iniciais da esclerose múltipla (EM). Revisão da literatura no tema, uma vez que muito poucos casos foram descritos no mundo.

**Métodos:**

Avaliação retrospectiva dos prontuários de pacientes com grave comprometimento cognitivo nos cinco primeiros anos de EM. Os resultados de testes neuropsicológicos e de imagens de ressonância magnética (RNM) são apresentados.

**Resultados:**

Quatro pacientes de diferentes departamentos de neurologia brasileiros foram avaliados, todos apresentando disfunção cognitiva muito grave que pode ser classificada como demência de rápida evolução. As imagens na RNM mostraram grave atrofia cerebral e lesões em gânglios da base em todas as pacientes.

**Conclusões:**

Embora muito rara, a alteração cognitiva grave na EM representa uma incapacidade importante e pode, em última análise, ser outra forma da doença.

## INTRODUCTION

After long periods with multiple sclerosis (MS), moderate to severe cognitive impairment is not an unusual finding in patients with the disease. In fact, cognitive dysfunction in long term MS is well recognized, being characterized by abnormalities in multiple domains of memory, speed of information processing, and executive function.^1^

A recent study by Staff et al.^2^ reported 21 cases of MS with severe cognitive impairment. In nine of the patients reported by these authors, the condition was marked by "fulminant" cognitive dysfunction early in the disease. The remaining patients also showed progressive signs of dementia, but not as intense as those in the nine cases cited. The patients, reported by group, typically exhibited psychiatric features, cerebellar syndrome and cortical signs and symptoms, such as seizures, aphasia, and apraxia. The majority of patients (14 out of 21) consumed tobacco. Brain magnetic resonance imaging typically demonstrated diffuse cerebral atrophy in these patients.

The purpose of this study was to report four additional cases of "fulminant" cognitive dysfunction from different MS study centers in Brazil.

## METHODS

The Research Ethics Committees of the academic institutions involved approved the publishing of retrospective medical records, provided patient identities were not disclosed. The respective caregivers of these patients previously agreed to the case report and signed a written consent term to this effect. The neuropsychological tests used for patient evaluation, neurological assessment, imaging and laboratory procedures, were all performed in accordance with the recommendations for MS diagnosis and follow up.

Data from retrospective evaluations of these patients were collected by the authors. The atypical presentation of MS in these patients led the investigators to proceed with extensive neuropsychological testing, as well as thorough diagnosis investigation for each case. All patients remain under the care of the same neurologist.

These patients developed progressive and severe cognitive symptoms in the early stages of the disease (within five years) as the most prominent feature of their MS. Other conditions were carefully considered in the differential diagnosis, and the final diagnosis of MS was established based on McDonald's criteria.^3^

Patients' response to immunomodulators for cognitive dysfunction was unremarkable, regardless of potential effects on relapse rates. Although some improvement was observed with use of immunosuppressive drugs, patients were only included if they required assistance to carry out activities of daily living.

Neuropsychological testing included the Mini Mental State Examination (MMSE), as well as cortical function tests for memory, praxia, speech, and gnosis. Executive function and visuo-spatial orientation were assessed by drawings. Fluency on digits and animal naming (Controlled Oral Word Association Test) were also tested.

## RESULTS

A summary of participants' demographic, medical, and neuropsychological status is given in Table 1.

**Table 1:** Summarized data on four MS patients at neuropsychological assessment.

	Case 1	Case 2	Case 3	Case 4
Gender	F	F	F	F
Present age	33	45	47	29
MS signs and symptoms	Cerebellum, cortex, optic nerve	Cerebellum, spinal cord	Cerebellum, optic nerve	Cerebellum
MS duration (years)	3	5	2	1
Number of relapses at the time of cognitive dysfunction	2	2	1	1
Follow-up time (years)	4	5	5	4
Clinical MS form	RRMS	RRMS	RRMS	RRMS
EDSS at the time of cognitive dysfunction	2.5	6.5	3.0	3.0
Schooling (years)	13	13	16	10
Smoker	no	yes	no	yes
Delusions, hallucinations	no	no	no	no
Childish behavior	yes	yes	yes	yes
Aware of cognitive dysfunction	no	no	yes (in the early stages)	yes (in the early stages)
**Severely compromised**				
Visual-spatial orientation (drawings)	yes	yes	yes	yes
Temporal orientation (anamnesis, MMSE)	no (in the early stages)	yes	yes	yes
Fluency tests (naming animals, numbers)	yes	yes	yes	yes
Memory and learning	yes	yes	yes	yes
MMSE score	14	9	12	14

RRMS: relapse-remitting multiple sclerosis. Patient 3, who had only one demyelinating episode at time of cognitive dysfunction onset, presented two further relapses. Her cognitive impairment remained on a progressive course; EDSS: expanded disability status scale 17. In all four patients, cerebellar symptoms were the main determinant of disability measured by EDSS.

All four patients were women, aged 26-43 years at the time of study, two of whom were smokers. Individuals had at least ten years' schooling, no history of illicit drug use, systemic or other neurological diseases. All participants had the relapsing-remitting form of MS.

One patient presented with severe and progressive cognitive impairment after the first relapse, while the other three patients manifested cognitive dysfunction following their second relapse.

Although all subjects had predominant cerebellar features, one case also had cortical involvement presenting as seizures, two cases presented optical neuritis, whereas another had spinal cord involvement with urinary symptoms. None of the patients presented with extrapyramidal signs and/or symptoms.

**Figure 1:**
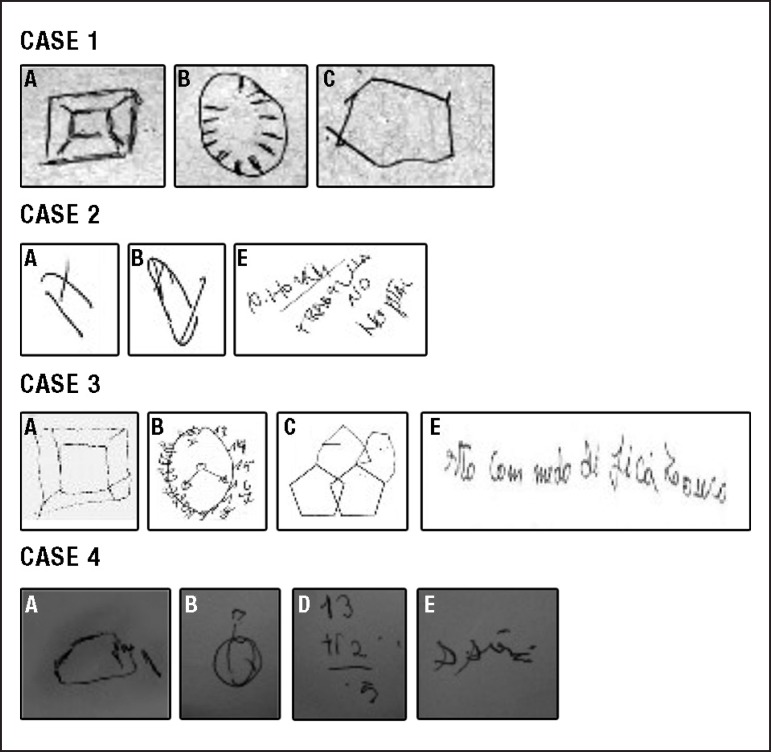
Drawings from neuropsychological evaluation of four MS patients. Drawing A = cube; B = clock; C = polygon intersection; D = simple arithmetic tasks; E = writing a sentence. Note: Case four was able to prepare the arithmetic task proposed (D) and started to perform a simple sum, but forgot what she was doing before completing the task.

Subjects' performance on neuropsychological tests was well below the expected values for relatively young, educated adults at the early stages of MS (Table 1 and Figure 1). Behavior of the patients was child-like, emotionally labile, and highly repetitive. Motor processing speed, speech, comprehension, praxia and gnosis (for example, facial recognition) were all moderately to severely compromised. Two patients were aware of their cognitive limitations while the other two did not grasp the severity of their condition. One patient showed preserved temporal orientation whereas the other three did not. In all patients, MMSE showed results characteristic of severe dementia (scores ≤14).

**Figure 2:**
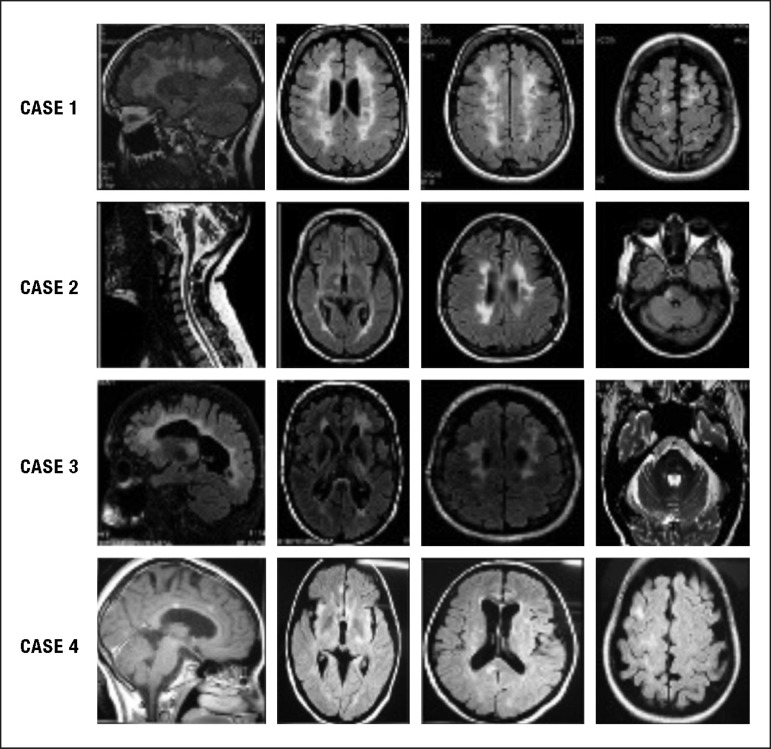
MRI of the four MS patients. Severe brain atrophy and basal ganglia lesions are evident in all four patients (MS history of less than 5 years).

Magnetic resonance imaging (MRI) showed accentuated degrees of brain atrophy and a large basal ganglia lesion in all four patients (Figure 2).

Some results on drawing tasks for these patients are depicted in Figure 1. Subjects' ability to draw a clock, a cube, and perform polygon intersection, sentence writing and simple arithmetic tasks were severely compromised.

## DISCUSSION

Cognitive impairment is known to be an important finding in MS. One fifth^4^ to one third^5^ of mildly disabled MS patients present a degree of cognitive deficit, while attention has been reported to be frequently altered in MS patients.^6^ Cognitive dysfunction may negatively impact coping strategies, worsening depression,^7^ while disability is reported to correlate with cognitive decline, apathy and depression.^8,9^ A recent meta-analysis on the subject of cognition and MS^10^ concluded that there is moderate cognitive decline in MS patients compared with healthy controls.

Regarding MRI findings, a recent report^11^ has shown correlation between periventricular lesions and cognitive deficit in MS, particularly related to psychomotor speed. Additionally, significant associations between cognitive impairment and lesions to interconnecting cortical regions thought to be involved in the processing of several cognitive domains have been reported.^12^ Despite some controversy on the matter, brain atrophy, particularly that of grey matter, seems to be the main marker for cognitive symptoms.^13^ Although the number and size of cortical lesions are debated in MS patients with cognitive deficit,^14^ it is generally agreed that such lesions play a major role in cognitive dysfunction. In our series, all four patients had cortical lesions and clinical manifestation of cortical involvement, with one subject presenting seizures and all four with dementia.

In summary, mild to moderate degrees of cognitive impairment involving several domains are often observed in later stages of MS, particularly among patients with brain atrophy and depression.

The patients reported belong to a different group to typical MS patients with cognitive dysfunction. Our patients are dementia cases with recently diagnosed MS.

Cerebellar signs associated to cognitive impairment in MS patients have been recently observed,^14,15^ but no data on severe dementia has been reported in this patient group.

No previous description of basal ganglia involvement in cases of dementia in early MS has been reported. Although this may be a fortuitous finding, it is important to register and study MRIs of other MS patients with these cognitive deficits. Corticobasal degeneration (CBD) was not a differential diagnosis in these cases of brain atrophy with basal ganglia lesions.^16^ The age group, absence of extrapyramidal signs and symptoms, predominant cerebellar syndrome and relapsing characteristics of our patients, are not consistent with CBD.

Another point of discussion is the elusive role of tobacco consumption in these patients. Staff et al.^2^ showed that two thirds of their MS cases with severe cognitive deficits were smokers, and the same prevalence was found in our group. As pointed out by these authors, this finding may possibly be correlated to a specific phenotype and this subject therefore warrants further investigation.

Despite being relatively rare, severe cognitive impairment with extensive brain atrophy in the early phase of MS represents an important disability and may ultimately constitute another form of the disease.
